# New Tricks for “Old” Domains: How Novel Architectures and Promiscuous Hubs Contributed to the Organization and Evolution of the ECM

**DOI:** 10.1093/gbe/evu228

**Published:** 2014-10-15

**Authors:** Graham Cromar, Ka-Chun Wong, Noeleen Loughran, Tuan On, Hongyan Song, Xuejian Xiong, Zhaolei Zhang, John Parkinson

**Affiliations:** ^1^Program in Molecular Structure and Function, Hospital for Sick Children, Toronto, Ontario, Canada; ^2^Department of Molecular Genetics, University of Toronto, Ontario, Canada; ^3^Department of Computer Science, University of Toronto, Ontario, Canada; ^4^Terrence Donnelly Centre for Cellular and Biomolecular Research, University of Toronto, Ontario, Canada; ^5^Banting and Best Department of Medical Research, University of Toronto, Ontario, Canada; ^6^Department of Biochemistry, University of Toronto, Ontario, Canada

**Keywords:** extracellular matrix, protein domains, domain architecture, evolution, domain networks

## Abstract

The extracellular matrix (ECM) is a defining characteristic of metazoans and consists of a meshwork of self-assembling, fibrous proteins, and their functionally related neighbours. Previous studies, focusing on a limited number of gene families, suggest that vertebrate complexity predominantly arose through the duplication and subsequent modification of retained, preexisting ECM genes. These genes provided the structural underpinnings to support a variety of specialized tissues, as well as a platform for the organization of spatio-temporal signaling and cell migration. However, the relative contributions of ancient versus novel domains to ECM evolution have not been quantified across the full range of ECM proteins. Here, utilizing a high quality list comprising 324 ECM genes, we reveal general and clade-specific domain combinations, identifying domains of eukaryotic and metazoan origin recruited into new roles in approximately two-third of the ECM proteins in humans representing novel vertebrate proteins. We show that, rather than acquiring new domains, sampling of new domain combinations has been key to the innovation of paralogous ECM genes during vertebrate evolution. Applying a novel framework for identifying potentially important, noncontiguous, conserved arrangements of domains, we find that the distinct biological characteristics of the ECM have arisen through unique evolutionary processes. These include the preferential recruitment of novel domains to existing architectures and the utilization of high promiscuity domains in organizing the ECM network around a connected array of structural hubs. Our focus on ECM proteins reveals that distinct types of proteins and/or the biological systems in which they operate have influenced the types of evolutionary forces that drive protein innovation. This emphasizes the need for rigorously defined systems to address questions of evolution that focus on specific systems of interacting proteins.

## Introduction

The emergence of complex, multicellular organisms required innovation of biological processes facilitating a variety of new structures and functions. In vertebrates, distinguishing features include specialized matrices such as cartilage, tendons, bones and teeth, and a pressurized vascular system. Technological advances in genetics and proteomics, begun over the past decade, continue to provide insights into the composition and organization of these processes (Gore et al. 2012; Onnerfjord et al. 2012; Simoes-Costa and Bronner 2013). At the same time, metazoan genome sequences are beginning to reveal evolutionary forces driving their genesis and subsequent refinement (Muller 1998; Huxley-Jones et al. 2007, 2009; King et al. 2008). Comparative studies of basal metazoans and their relatives, for example, suggest that homologs of many of the genes that drove the innovation of multicellularity arose in free-living ancestors of metazoans (King et al. 2008). These include several members of the extracellular matrix (ECM), a fundamental metazoan innovation with central roles in a variety of diverse functions including: Respiration, feeding, reproduction, locomotion, osmoregulation, hemostasis, and cognition (Di Lullo et al. 2002; Ozbek et al. 2010; Hynes 2012). However, beyond their origins, the subsequent evolutionary forces that guided the development of systems such as ECM remain largely unexplored.

The human ECM comprises approximately 324 proteins that self-organize into a complex array of fibers and ancillary proteins, providing essential scaffolding for arranging cells into tissues (Chautard et al. 2011; Cromar et al. 2012; Hynes and Naba 2012). The ECM is also involved in morphogenesis, through the mechanical regulation of cells and intercellular junction positioning (Tseng et al. 2012), and acts as a sink for a variety of growth factors allowing the system to rapidly respond to spatially relevant signals (Taipale and Keski-Oja 1997; Hynes 2009). In earlier work (Cromar et al. 2012), we have shown that, while one-third of human ECM genes are shared with other metazoans, the remaining two-thirds appear to be recent vertebrate-specific innovations. What are not clear are the mechanisms that underpin these innovations and how the organization of protein domains, as fundamental units of selection, contributed to ECM evolution.

Aside from evolutionary studies focusing on individual gene families (e.g., [Aouacheria et al. 2004; Ewan et al. 2005; Huhtala et al. 2005; Huxley-Jones et al. 2005; McKenzie et al. 2006]), few studies have attempted to examine the evolution of the ECM as a complete system. In one study of 60 ECM genes from *Ciona*, the authors concluded that the increased complexity observed in vertebrate ECM proteins was largely driven by large-scale duplications of preexisting genes, (Huxley-Jones et al. 2007). Subsequent reviews have compared ECM genes in various taxa, linking the occurrence of domain gains in selected proteins with their potential functional adaptations (Hynes 2012; Hynes and Naba 2012). Indeed, although proteins containing only a single domain are in the minority, vertebrate proteins, particularly those targeted to the extracellular milieu, are enriched for multidomain architectures (Bork et al. 1996; Hohenester and Engel 2002; Apic et al. 2003; Buljan et al. 2010; Zmasek and Godzik 2012). To date, analyses of domain evolution in ECM proteins have been largely restricted to the functional annotation of particular three-dimensional domain structures (Bork et al. 1996; Hohenester and Engel 2002). Increasing numbers of metazoan genomes as well as the recent systematic curation of ECM proteins now provide the opportunity to extend these largely descriptive studies to explore the principles of ECM domain architectural evolution relative to other vertebrate proteins.

At the whole-proteome level, recent studies suggest that domain gain and loss events, occurring most commonly at the C-terminus, dominate and that transposition, exon shuffling, and recombination appear to play only minor roles (Apic et al. 2003; Moore and Bornberg-Bauer 2012; Bornberg-Bauer and Alba 2013; Moore et al. 2013). However, it is not clear if the relative contribution of these events is consistent across the entire proteome or whether distinct types of proteins and/or the biological systems in which they function, influences the types of evolutionary forces that drive protein innovation. It has been assumed, for example, that exon shuffling is a predominant factor in ECM protein evolution due to the fact that most ECM domains are encoded as exonic units (Hynes 2012). Furthermore, previous investigations of domain architectures have been limited to the study of domain pairs or triplets, neglecting the possible conservation of architectures representing higher orders of domains (HOODs).

Characterized by large, multidomain proteins with distinct physical and functional characteristics, the ECM represents an ideal system to explore how patterns of domain evolution may vary for a given class of functionally related proteins. Here, we present a systematic study of domain gain, loss, and rearrangement events that have contributed to human ECM innovation and highlight how the evolution of domain architectures has resulted in the emergence of vertebrate innovations.

## Materials and Methods

### Source for Proteins

The list of ECM components correspond to a previously defined list of structural and soluble components, exclusive of membrane-associated proteins (Cromar et al. 2012) (supplementary spreadsheet S1, Supplementary Material online). To identify orthologs of these components, protein sequences for 131 fully sequenced eukaryotic genomes (species and phylogeny described in supplementary fig. S1 and spreadsheet S2, Supplementary Material online) were obtained as previously described (Xiong et al. 2011). Conservation of proteins was determined using the longest peptide associated with each human ECM gene (termed the “reference”). Orthologs and paralogs (collectively referred to as the “targets”) were detected using Inparanoid as previous (Berglund et al. 2008; Xiong et al. 2011). Protein conservation profiles were clustered using Cluster 3.0 (de Hoon et al. 2004).

### Source for Domains

ECM-associated domains were defined as those detected in at least one of the human ECM proteins described above. Domain predictions were performed at the level of the whole proteome, across all 131 species on a parallel computing platform using profile hidden Markov models (HMMER 3.0 [Eddy 2011] with default parameters as implemented in PfamScan [Punta et al. 2012]). Data flow was handled in a data processing pipeline written in house using Perl and results were stored and manipulated using PostgreSQL. We rely on Pfam-defined domains (Punta et al. 2012) which while subject to biases in the choice of organisms to generate seed alignments for the definition of domains, nonetheless provide a well-established framework to study domain evolution. Our analysis included curated Pfam-A “domains” and “families” where a domain is defined by Pfam as a “structural unit” and a family is defined as “a collection of related protein regions.” Note: We chose to exclude “motifs” and “repeats” as defined in Pfam because they did not meet the criteria for domains as independent folding units.

### Domain Enrichment

The frequency of each domain in the human proteome and in the ECM subset was calculated using Perl scripts developed in house based on the PfamScan domain predictions described above. Domain architectures were preprocessed to remove large tandem duplications (i.e., greater than two domains) which would otherwise inappropriately skew domain frequencies. This was done by iteratively removing duplicated domains until only a single (A-A) pair remained. Proteins were then classified as either single or multidomain depending on their domain content. Domains were classified as appearing in single or multidomain proteins or both. Domain enrichment was determined using the hypergeometric test with false discovery rate.

### Conservation of Domains and Domain Pairs

Domains found in human ECM reference sequences were compared against the full proteome of each species. As above, domain architectures were preprocessed to remove tandem duplications which would otherwise inappropriately weight domain frequencies. Domain pairs refer to immediately adjacent domains. Domain pairs were defined in the N- to C-terminal direction and reverse orientations were considered to be unique (i.e., A-B ≠ B-A). A domain or domain pair was considered to be conserved if it appeared in at least one of the proteins in a given species. Patterns of domains and domain-pair conservation were hierarchically clustered using Cluster 3.0 grouping them into blocks representing similar conservation profiles.

### Conservation of Domain Architecture

The domain arrangement for each reference sequence was compared with each of its corresponding orthologs (the longest paralog where multiple paralogs were identified). Tandem duplications were included in this analysis. Domain gains, losses, gains and losses of domain repeats, and complex rearrangements, were identified based on the most parsimonious change (all comparisons being relative to the human reference sequence). Complex rearrangements were those involving combinations of gain and loss events. Domain architecture conservation profiles were hierarchically clustered using Cluster 3.0.

### Tandem Repeats

We assessed the total number of Pfam-A domains in each human ECM protein and the relative contribution of “first instance domains” (FIDs) and “non-FIDs” (NFIDs) using a simple counting method. The first occurrence of a domain was counted as an FID and any subsequent occurrence of the same domain as a NFID. By definition, the occurrence of NFIDs is highly correlated with tandem repeats and for our purposes we assume they are equivalent with the caveat that some NFIDs could be the result of two or more occurrences of the same domain separated by one or more other domains. For example, an alternating pattern such as ABABA would be recorded as having three NFIDs and algorithmically we would not distinguish this from AAABB—which has bona fide tandem repeats. To mitigate this source of error, we only considered proteins with a larger number of NFIDs than FIDs to contain tandem domain repeats. To quantify the association between late domain gains and NFIDs, we classified proteins according to their domain architecture conservation patterns as having undergone prevertebrate (“early”) or postvertebrate (“late”) domain gains and whether or not those gains were associated with tandem repeats (NFIDs > FIDs) (supplementary spreadsheet S1, Supplementary Material online). Enrichment for late domain gains was assessed via hypergeometric test with Bonferroni correction.

### Domain Alignment

To provide visualizations of domain architectures for reference proteins across species, we performed domain alignments as follows: For each gene, we tokenized each homolog as a sequence of domains where domains were assigned an arbitrary letter code in sequence. For example, the first domain is assigned the letter “A,” the next unique domain the letter “B” and so forth. Thereafter, recurrences of domains within the same protein or within the same set of paralogs were assigned the corresponding letter code (e.g., ABCAA). The resulting short strings of letters were rapidly aligned using a custom Perl script implementing the BioPerl SimpleAlign module with ClustalW as the alignment method. The ktuple parameter was set to 2 (“ktuple” ≥ 2) and we used an identity substitution matrix (“matrix” ≥ “ID”).

### Domain Adjacency Network

Using the ordered (N- to C-terminal) arrangement of domains occurring in our reference proteins, we constructed a list of domain pairs occurring in the human ECM and their orthologs from each species and used them to construct a network of domain adjacency. The statistical significance of domain pairs was determined by comparing the frequency of each pair in the real human proteome with that of 10,000 simulated proteomes (“Bootstrap resampling”—see below). The reported *P* value represents the number of times out of 10,000 simulations that a given pair was found as frequently as or more frequently than in the real proteome by chance alone. The corresponding *z*-scores were used to weight the edges of the subnetwork of all human pairs and clustered using the Markov Cluster Algorithm (MCL) (Enright et al. 2002) with a default inflation value of 2.1 to predict putative domain modules. To avoid the inclusion of negative edge weights not handled by MCL, the set of *z*-scores was transformed by addition of a small positive value such that the lowest value became zero. Pfam to G.O. mappings (Hunter et al. 2009) were used to associate functional annotations with each domain. Modules were then annotated according to the most frequent term common to the domains within the module as visualized in WordCloud (Oesper et al. 2011). Overlap between domain modules and protein–protein interaction (PPI)-based modules (the latter defined and annotated in our previous study) was accomplished by converting proteins within modules to their corresponding domain representation. Protein modules and domain modules with the largest number of overlapping domains were matched for the purpose of transferring annotations. To assess the significance of domain overlaps between domain and PPI-based modules the occurrence of domain overlaps in “real” pairs were compared with the overlaps of the highest overlapping module in each of 10,000 randomized networks constructed with the same domain distribution. Networks were visualized using Cytoscape (Shannon et al. 2003).

### Domain Promiscuity

We adopted the weighted bigram frequency used by Basu (Basu et al. 2008) as a measure of domain promiscuity ($\pi_{i}$). This was originally derived from the Kullback–Leibler information gain formula:

(1) $$\pi_{i}={\text{β}}_{i}\times \log\left(\frac{{\text{β}}_{i}}{f_{i}}\right)$$

The bigram frequency${\text{β}}_{i}$ is:

(2) $${\text{β}}_{i}=\frac{T_{i}}{\frac{1}{2}\sum_{j=1}^{t}T_{j}}$$

where *t* is the number of distinct domain types. $T_{i}$ is the number of unique domain neighbors of domain *i* and $f_{i}$ is the frequency of domain *i* in the genome, calculated as $n_{i}/N$, where $n_{i}$ is the total count of domain *i* and *N* is the total number of domains detected in the given genome:

(3) $$N=\sum_{i=1}^{t}n_{i}$$

Note $\pi_{i}$ is influenced by the number of network neighbors as well as by the number of detected domains. The metric is therefore unsuitable for direct comparison of promiscuity scores between studies with different underlying domain sets. Promiscuity scores were validated through rank comparisons with a previously generated set (Basu et al. 2008). To determine the relative occurrence of promiscuous domains among network hubs and nonhubs in the previously published PPI-based network (Cromar et al. 2012), we defined hubs as proteins having degree ≥5, consistent with previous studies (Han et al. 2004; Kim et al. 2006; Patil et al. 2010a).

### HOOD Architectures

A frequent sequential pattern can be defined as an ordered set of domains found in at least *n* proteins (support = *n*). For example, the sequential pattern (A,B,C) can be found in proteins with domain architectures: (A,B,C,D), (X,A,B,C), (Y,A,Y,B,C), (X,Y,A,Z,B,B,C). It should be noted that the pattern can be discontinuous as long as the ordering is preserved (in the example, A is followed by B which is then followed by C). We used PrefixSpan (Jian Pei et al. 2001) to find frequent sequential patterns in human ECM reference proteins and their orthologs in nine species representing basal metazoa/metazoa: *Monosiga brevicollis*, *Amphimedon queenslandica*, *Hydra magnipapillata*, *Caenorhabditis elegans*, *Drosophila melanogaster*, *Ciona intestinalis*, *Danio rerio*, *Xenopus tropicalis*, and *Gallus gallus.* Input files consisted of unprocessed domain architectures (i.e., including domain repeats) representing the presumed orthologs of the reference sequence (longest inparalogs). Because the presence of highly related sequences would tend to inflate the occurrences of patterns found in, for example, similar splice variants, the sequences were prefiltered to remove redundant sequences (above 90% similarity) prior to pattern analysis. Thresholds of 90%, 95%, and 97% are commonly used to filter out redundant sequences in taxonomic studies (Mohamed and Martiny 2011), whereas Uniprot reference clusters (Suzek et al. 2007) use cutoffs of 90% and 50%. Here, using 90% and 50% cutoffs resulted in similar number of nonredundant sequences implying that a 90% similarity cutoff was sufficient to remove paralogous sequences. Calculation of percent similarity was based on BLAST output:

(4) $$\%\text{Similarity}=\frac{\left(\text{\%Id × Match region}\right)}{\text{Length}}$$

In implementing PrefixSpan we set the only parameter, support, as 3 such that a frequent sequential pattern is one that is found in at least three proteins. Because the inclusion of domain repeats led to exponential memory usage, for practical purposes we limited output to patterns involving four domains or less.

Domain patterns were hierarchically clustered using Cluster 3.0 grouping similar conservation patterns. The frequency of domains within conservation groups was visualized using WordCloud (Oesper et al. 2011). Domain patterns were organized using the Enrichment Map plug-in for Cytoscape (Merico et al. 2010) into clusters representing related patterns with overlapping sets of domains. Statistical significance of domain patterns was determined by comparing the frequency of each pattern in the real human proteome with that of 10,000 simulated proteomes (see Simulated Proteomes). The reported *P* value represents the number of times out of 10,000 simulations that a given pattern was found as frequently as or more frequently than in the real proteome by chance alone.

### Simulated Proteomes

Simulated proteomes were generated to assess the significance of observed domain pairs and patterns relative to their occurrence at random. First, using Pfam-A domain predictions for the complete human proteome we precalculated domain frequencies and domain distributions (number of domains in each protein) in the real proteome. To populate each simulated proteome, we constructed a set of “pseudo-proteins” by randomly selecting domains (without replacement) from a pool reflecting the domain frequencies of the real human proteome. As domain pairs were created in the growing pseudo-proteins, the pair was propagated across eligible pseudo-proteins a random number of times before individual domain selection resumed. Individual domains propagated as pairs continued to be removed from the domain pool during this process. If the availability of either domain in the pair was exhausted in the domain pool or if the random propagation limit for that pair was reached, the propagation of that pair ceased and individual domain selection was resumed. This process was continued until all domains in the pool were exhausted. For domain pairs, simulated proteomes were constructed using domain frequencies corresponding to the preprocessed domain architectures of human ECM proteins (i.e., without domain repeats), and the random placement of domains was constrained so as to prevent the random creation of tandem domain repeats. Random domain pairs resulting from these simulations therefore reflected the conditions used to evaluate domain pairs in the real proteome. As sequential pattern mining was performed on unprocessed domain architectures, simulated proteomes created for assessment of HOODs were not constrained in this way.

## Results

### Evolution of the ECM is Driven in Part by the Invention of Novel Domains

**Fig. 1.—:**
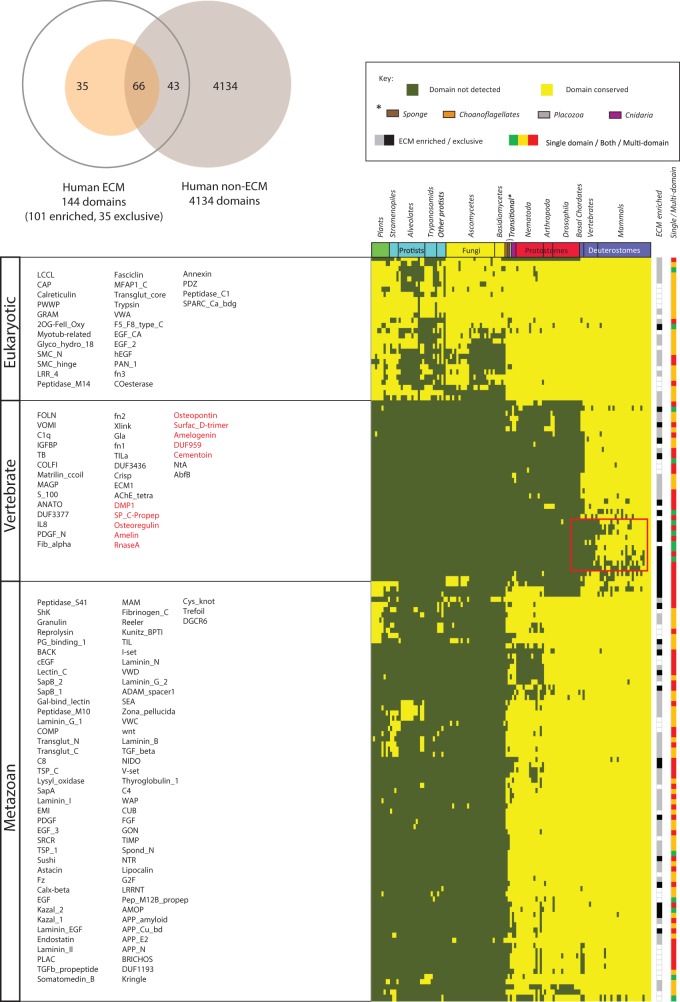
Conservation of ECM domains highlighting three broad groupings corresponding to domains of eukaryotic, metazoan, and vertebrate origin. (*A*) Relative numbers of ECM, ECM-enriched, and ECM exclusive domains relative to the total number of Pfam-A domains detected in humans. (*B*) Domains occurring in ECM proteins across 131 species are represented as colored tiles (present = yellow tiles; absent = olive tiles). Domain names are arranged in order from top to bottom and left to right. Species are arranged according to established phylogenetic relationships, and domains were hierarchically clustered (city block, average linkage) into groups representing similar conservation patterns. The track to the right of the heatmap indicates domains that are significantly enriched in ECM proteins (*P* < 0.05 by hypergeometric test with correction for false discovery rate) and those exclusive to ECM proteins (gray vs. black). Note that most of the ECM-enriched domains are of early metazoan origin (i.e., conserved with protostomes), despite the fact that two-thirds of human ECM *proteins* do not have detectable orthologs outside the deuterostomes (Cromar et al. 2012). A cluster of ECM-exclusive domains (boxed red) may have been associated with skeletal reorganization during and subsequent to the fish–tetrapod transition. A second color track indicates domain participation in single/both/multidomain arrangements (green/yellow/red). Additional details are provided in supplementary spreadsheet S7, (Supplementary Material online).

In this section, we establish the domain repertoire of ECM proteins and, examine their origins and the contribution of novel domains to novel ECM functions. Using domain definitions as provided by the curated Pfam-A resource (see Materials and Methods), we find that of 4,243 domains in the human proteome, 144 ( ∼ 3.4%) are associated with ECM proteins, of which 101 are significantly ECM-enriched (*P* < 0.05, Hypergeometric test with false discovery rate correction). Among the enriched domains, 35 appear exclusive to ECM proteins (table 1 and fig. 1*A*) and supplementary spreadsheets S3–S6, Supplementary Material online). To assess the origins of these 144 domains, we examined their occurrence within the genomes of 131 fully sequenced eukaryotes. Three distinct groups are apparent, corresponding to domains with eukaryotic, metazoan, and vertebrate origins (fig. 1*B*) and supplementary spreadsheet S7, Supplementary Material online). Approximately four out of every five ECM domains (79.2%) are conserved but specific to choanoflagellates and metazoans, suggesting that the emergence and subsequent evolution of the ECM involved the recruitment of both a limited number of ancestral, premetazoan domains (20.8%), together with the innovation of a larger number of novel domains, prior to the branching of the various metazoan lineages.

**Table 1:** Pfam-A Domains Found Exclusively in ECM Proteins (human proteome)

Pfam ID	Domain Name	Description
PF03146	NtA	Agrin NtA domain
PF05270	AbfB	Alpha-L-arabinofuranosidase B (ABFB)
PF05111	Amelin	Ameloblastin precursor (Amelin)
PF02948	Amelogenin	Amelogenin
PF11598	COMP	Cartilage oligomeric matrix protein
PF06482	Endostatin	Collagenase NC10 and Endostatin
PF01413	C4	C-terminal tandem repeated domain in type 4 procollagen
PF07263	DMP1	Dentin matrix protein 1
PF11857	DUF3377	Domain of unknown function (DUF3377)
PF11918	DUF3436	Domain of unknown function (DUF3436)
PF06121	DUF959	Domain of unknown Function (DUF959)
PF05782	ECM1	ECM protein 1 (ECM1)
PF07474	G2F	G2F domain
PF08685	GON	GON domain
PF00396	Granulin	Granulin
PF00052	Laminin_B	Laminin B (Domain IV)
PF06008	Laminin_I	Laminin Domain I
PF06009	Laminin_II	Laminin Domain II
PF00055	Laminin_N	Laminin N-terminal (Domain VI)
PF09006	Surfac_D-trimer	Lung surfactant protein D coiled-coil trimerisation
PF00413	Peptidase_M10	Matrixin
PF05507	MAGP	Microfibril-associated glycoprotein (MAGP)
PF00865	Osteopontin	Osteopontin
PF07175	Osteoregulin	Osteoregulin
PF03572	Peptidase_S41	Peptidase family S41
PF01471	PG_binding_1	Putative peptidoglycan binding domain
PF01549	ShK	ShK domain-like
PF06991	Prp19_bind	Splicing factor, Prp19-binding domain
PF06468	Spond_N	Spondin_N
PF08999	SP_C-Propep	Surfactant protein C, N-terminal propeptide
PF00683	TB	TB domain
PF05735	TSP_C	Thrombospondin C-terminal region
PF00965	TIMP	Tissue inhibitor of metalloproteinase
PF10511	Cementoin	Trappin protein transglutaminase binding domain
PF03762	VOMI	Vitelline membrane outer layer protein I (VOMI)

Among the ancestral, premetazoan domains recruited to the ECM (fig. 1*B*) are several that are ECM-enriched as well as one apparently exclusive to the ECM in humans; MFAP1_C (PF06991) is a conserved C-terminal domain of human MFAP1 (microfibrillar-associated protein), an important component of elastin-associated microfibrils (Horrigan et al. 1992; Liu et al. 1997; Hann and Fautsch 2011; Baldwin et al. 2013). Interestingly, ongoing Pfam curation efforts recently uncovered evidence implicating the corresponding yeast ortholog (PRP19) in pre-mRNA splicing (Ren et al. 2011). Human MFAP1 has been isolated in spliceosomal fractions, suggesting retention of its premetazoan function and highlighting the ability of apparently established domains to acquire additional, diverse functions.

The distribution of ECM-enriched and ECM-exclusive domains across all three evolutionary periods suggests domains arise through a continual process yielding opportunities to develop novel functionalities. For example, the process of skeletonization began early in the vertebrate lineage corresponding to the transition from jawless to jawed vertebrates and continued with gradual increasing complexity and rearrangements in the four skeletal tissues (bone, dentin, enamel, and cartilage) (Donoghue and Sansom 2002; Donoghue et al. 2006). Corresponding to this, in addition to a few metazoan-specific domains that have acquired roles in vertebrate skeletonization (e.g., COLFI; PF01410, PDGF; PF00341), there are a number of vertebrate-specific domains with obvious connection to skeleton formation (e.g., MATRILIN; PF10393, MAGP; PF05507, PDGF_N; PF04692, FN1/2; PF0039/40, Gla; PF00594) as well as a smaller subgroup of more recent origin (fig. 1*B* boxed and supplementary spreadsheet S7, Supplementary Material online). The latter are enriched in ECM-exclusive domains associated with proteins involved in biomineralization (e.g., DMP1; PF07263, Osteoregulin; PF07175) (Narayanan et al. 2003; Quarles 2003), enamel (e.g., Amelin; PF05111 and Amelogenin; PF02948) (Li et al. 2001; Shintani et al. 2002), and bone remodeling (Osteopontin; PF00865) (Reinholt et al. 1990). Given their likely role in feeding, respiration, and locomotion, the emergence of these latter domains after the split between fish and tetrapods is at least partly responsible for the transition and further adaptation to a land-based lifestyle (Ahlberg et al. 2008; Downs et al. 2008).

Having established a list of ECM-associated domains, their origins and the role of novel domains in establishing new functions, in the next section we consider the tendency for domains in the ECM to form stable combinations with other domains (a trait referred to as “promiscuity”), and its potential role in the context of a previously defined network of ECM protein interactions (Cromar et al. 2012).

### Organization of the ECM is Mediated by a Relatively Small Number of Highly Promiscuous Domains

Previous studies have found extracellular proteins possess a relatively high incidence of promiscuous domains relative to most other proteins (Basu et al. 2008, 2009). To examine if this is also a general feature of ECM proteins, we used a weighted bigram frequency metric (Basu et al. 2008), which normalizes for domain frequency, to define the relative promiscuity of domains appearing in multidomain proteins (2,282/4,243) in humans (table 2 and supplementary spreadsheet S8, Supplementary Material online). Domains were grouped into three age categories based on their conservation across the Eukarya (E), Metazoa (M—including transitional metazoan and choanoflagellates) or, Vertebrata (V). Of the three age categories, vertebrate-derived domains were characterized by domains of low frequency and promiscuity while metazoan and eukaryotic domains, included domains with higher frequency and/or higher promiscuity (supplementary fig. S2 and spreadsheet S9, Supplementary Material online).

**Table 2:** Top 30 Promiscuous Domains

Domain	Name	Direct Neighbors	Cooccurrence	Weighted Bigram Frequency	Found in ECM	Enriched in ECM	Rank^a^
PF00595.18	PDZ	52	71	0.021795689			9
PF00169.23	PH	50	77	0.014169779			2
PF00018.22	SH3_1	39	56	0.011726788			3
PF00627.25	UBA	18	26	0.011594278			38
PF00533.20	BRCT	15	26	0.010918809			21
PF00397.20	WW	20	24	0.010536495			34
PF00628.23	PHD	27	42	0.01048959			4
PF00004.23	AAA	20	22	0.009510768			1
PF12796.1	Ank_2	52	74	0.009320291			7
PF00008.21	EGF	32	51	0.009110883	Y	Y	28
PF00641.12	zf-RanBP	13	16	0.00850045			No match
PF07653.11	SH3_2	23	37	0.007659358			No match
PF07699.7	GCC2_GCC3	10	17	0.007279206			No match
PF00536.24	SAM_1	20	28	0.007075622			24
PF00093.12	VWC	14	20	0.006970266	Y	Y	63
PF07647.11	SAM_2	15	26	0.006860156			No match
PF07648.9	Kazal_2	15	28	0.006860156	Y	Y	98
PF00130.16	C1_1	17	25	0.006780639			8
PF00788.17	RA	15	26	0.006563627			31
PF07714.1	Pkinase_Tyr	27	43	0.006378603			20
PF00013.23	KH_1	14	17	0.006332245			147
PF00620.21	RhoGAP	19	27	0.006246943			18
PF00791.14	ZU5	9	10	0.006195204			159
PF00787.18	PX	16	23	0.006141415			14
PF00226.25	DnaJ	16	17	0.006141415			30
PF00610.15	DEP	11	18	0.006089229			43
PF07645.9	EGF_CA	32	49	0.005995208	Y	Y	25
PF00629.17	MAM	10	16	0.005971257	Y		68
PF01585.17	G-patch	12	14	0.005902413			80
PF00621.14	RhoGEF	19	32	0.005827127			No match
PF00092.22	VWA	21	31	0.005791992	Y	Y	67

^a^Top 30 Promiscuous domains in the human proteome based on weighted bigram frequency along with their rank in a similar list of 215 highly promiscuous domains in eukaryotes based on mean promiscuity (π) value over 28 species (Basu et al. 2008).

On the basis of the top ten percentile of promiscuity scores (i.e., the promiscuity cutoff for the 90th percentile), we defined a weighted bigram frequency of 0.0021 as a cutoff for “high promiscuity” (supplementary fig. S3 and spreadsheet S10, Supplementary Material online). Of the 124 ECM-associated domains that appear in multidomain proteins, we found 38 (30.6%) could be defined as highly promiscuous which represents a significant enrichment compared with non-ECM associated domains at 224/2,282 (9.8%) (*P* < 1.5 × 10^−^^4^, Hypergeometric test with Bonferroni correction). Overall, as the remaining 86/124 ECM-associated domains (including the majority of domains of vertebrate, metazoan, and eukaryotic origin) were of low promiscuity, we did not find a statistically significant association between promiscuity and domain age (*P* > 0.05, chi-square goodness of fit test, supplementary spreadsheets S9–S10, Supplementary Material online). Given the sigmoidal distribution of promiscuity scores (supplementary fig. S4, Supplementary Material online), we conclude that promiscuity is not a general characteristic of ECM domains, but rather, restricted to a small subset of ECM domains in which high promiscuity likely plays some functionally important role.

Domains, via protein binding, play a fundamental role in the organization of protein-interaction networks with multidomain proteins often representing “hubs” (Patil et al. 2010a, b). To examine the relationship between promiscuous domains and protein hubs within our previously defined network of ECM proteins, we considered 173/324 ECM proteins with known interactions and defined domain architectures (supplementary spreadsheet S11, Supplementary Material online). We found that hubs (defined as having a node degree ≥5) are significantly enriched in highly promiscuous domains (*P* < 0.05, Bootstrap resampling). More intriguingly, network hubs are significantly enriched in structural proteins (38/92 hubs, *P* < 0.05, Bootstrap resampling) and these structural proteins, which accounted for 60/173 proteins, were significantly enriched in promiscuous domains (31/60, *P* < 0.005, Bootstrap resampling).

Consistent with the proposed role of domains in network connectivity, the three highest promiscuity domains in humans (PDZ, EGF, and VWC; PF00595, PF00008, and PF00093, respectively; table 2), are all highly conserved and facilitate PPIs, functioning in both signaling and structural contexts. Although PDZ is likely not a bona fide ECM domain (see Discussion) the other two domains are ECM-enriched. Together, the preceding suggests that preferential occurrence of promiscuous domains in hubs is a critical determinant of PPI network topologies; a property that, for the ECM, coincides with the tendency for these hubs to be structural. Given the importance of promiscuous domains and hence multidomain architectures in organization of the ECM protein interaction network, we were next interested in examining the evolutionary dynamics of multidomain arrangements and the contribution of domain gain, loss, and rearrangement events on lineage-specific innovations.

### Domain Gain is a Major Driving Force for ECM Innovation in the Human Lineage

**Fig. 2.—:**
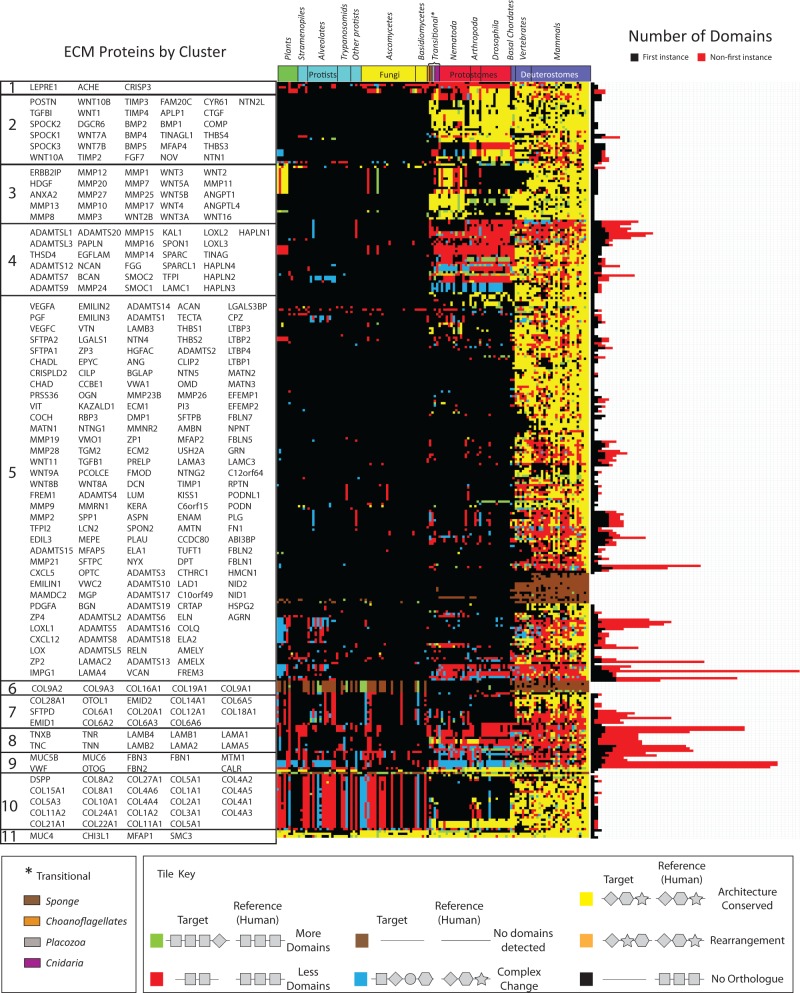
Conservation of ECM architectures revealing high-level, clade-specific patterns of domain gain, loss, and rearrangement among the orthologs of human ECM proteins. Each colored tile in the heatmap (center) represents the domain composition of a protein in a given species relative to the corresponding human reference ortholog. Differences in domain composition or arrangement have been color coded with, for example, fully conserved architectures in yellow (see color key). Proteins were hierarchically clustered (city block method, average linkage) into groups representing similar conservation profiles. Orthology was determined using a previously published Inparanoid-based method (Xiong et al. 2011) using the longest peptide sequence associated with the corresponding gene. Domain composition was based on detection of Pfam-A families using the highest scoring ortholog to the human reference sequence. The phylogeny represents 131 fully sequenced eukaryotes arranged according to established phylogenetic relationships. For each protein, the total number of domains is plotted as a stacked bar graph (right), where the number of FI domains is shown in black and the number of NFI domains in red. See also supplementary spreadsheet S1, Supplementary Material online.

To obtain a global overview of domain gain and loss events, we generated domain architectures for orthologs of ECM proteins identified in 131 fully sequenced eukaryotic genomes. We found that across 33 deuterostome genomes, 62.8% of ECM orthologs had identical domain architectures, suggesting selective pressure to maintain architecture (fig. 2 and spreadsheet S1, Supplementary Material online). Where we observed changes in human ECM protein domain architecture, domain gain was found to be more common than domain loss (28.9% vs. 5.2% of orthologs) (supplementary spreadsheet S12, Supplementary Material online).

Domain rearrangements, representing the shuffling of otherwise identical domain complements, were rare (0.2%), whereas more complex changes (combinations of gain and loss events) were relatively rare (2.8% of orthologs), as consistent with previous global domain studies (Marsh and Teichmann 2010). As expected, the protostomes exhibit a drop in conservation of domain architectures (34.3% of orthologs), resulting from an increase in both domain gains and losses in human ECM proteins relative to their protostome orthologs (44.2% and 7.5%, respectively), together with a relatively high number of more complex changes (13.6%) (supplementary spreadsheet S12, Supplementary Material online). Thus, as for other systems (Bornberg-Bauer et al. 2010; Toll-Riera and Alba 2013), domain losses are more likely to be deleterious to ECM function than domain gains, which have the potential to provide additional lineage-specific adaptations.

**Fig. 3.—:**
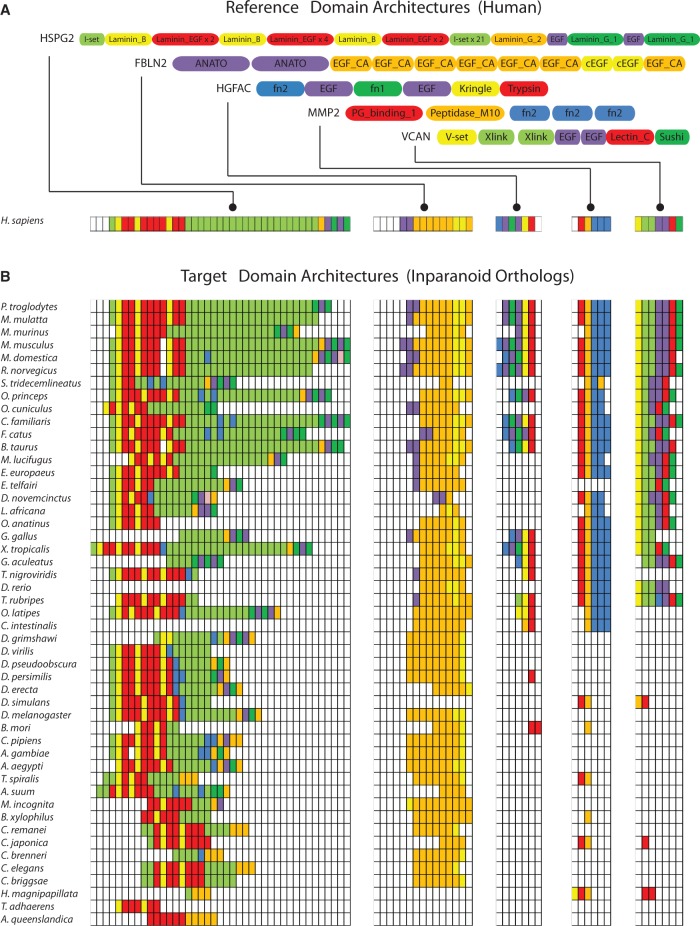
Sample domain architectures illustrating their relative conservation across 131 species with gains and losses of domains occurring throughout. (*A*) Domain arrangements of human HSPG2, FBLN2, HGFAC, MMP2, and VCAN based on Pfam-A. (*B*) Corresponding domain based alignments of homologous proteins as detected using Inparanoid. Homologs are arranged in phylogenetic order with mammals at the top. Species with no detectable ortholog are not shown. Full domain alignments for each protein are shown in supplementary spreadsheets S13–S17, Supplementary Material online.

Proteins comprised of a majority of NFIDs, associated with tandem domain repeats (see Materials and Methods), were enriched for recent (vertebrate) domain gains (*P* < 0.05, hypergeometric test with Bonferroni correction), whereas prevertebrate domain gains corresponded with low numbers of domain repeats (i.e., gain of novel domains). Interestingly, repeats were enriched within structural proteins (*P* < 0.005, chi-square goodness of fit test, fig. 2 and supplementary spreadsheet S1, Supplementary Material online). Despite an appearance that recent domain gains occur solely at the divergence of primates from other mammals as might be inferred for groups 4, 5, 7, 8, and 9 in figure 2, domain alignments of individual proteins (fig. 3 and supplementary spreadsheets S13–S17, Supplementary Material online), revealed that gains (as well as losses) occur throughout the deuterostome lineage. For example, perlecan (HSPG2) is composed of a conserved core of Laminin B and Laminin EGF domains, supplemented with increasing numbers of I-set domains. Fibulin-2 (FBLN2) has acquired an ANATO domain (PF01821) initially detected in fish and subsequently duplicated in mammals. Finally, hepatocyte growth factor activator (HGFAC) demonstrates a mosaic of domain gains and losses throughout the deuterostome lineage.

Consistent with previous studies of domain evolution, domain gain during ECM evolution appears to be more important in driving innovation than domain loss. Compared with all human proteins, in which vertebrate-specific domains have been estimated at 12.3% (426 of 3,465) (Toll-Riera and Alba 2013), we find that 24.3% (35 of 144) of domains found in ECM proteins are vertebrate-specific. Nevertheless, the innovation of a proportionally larger number of vertebrate-specific ECM proteins (two-third of human ECM proteins) suggests the involvement of additional mechanisms. In the next section, we explore the contribution of novel domain architectures to ECM evolution.

### Novel ECM Protein Domain Architectures Are Largely Age-Independent

The recruitment of additional domains to the ECM, in addition to providing intrinsic functionality, offers the potential to derive new functions through combining with other domains. Compared with other human domains, ECM domains are more often associated with multidomain proteins (*P* < 0.01 χ^2^ test; supplementary fig. S5, Supplementary Material online). Of the 144 ECM domains, 62 (43%) are found exclusively in multidomain proteins, whereas an additional 67 (46.5%) are found both in single and multidomain proteins. This compares with 1,665 (39.2%) and 775 (18.3%), respectively, for non-ECM domains. In general, ECM domains rarely occur *only* in single-domain architectures (15/144 [10.4%]) though when they do, tend to be of recent origin. For example, 5/10 of the highlighted, tetrapod-specific domains are found exclusively in singleton architectures (fig. 1*B*) though the other five occur exclusively in multidomain proteins. Together these findings suggest that when new ECM domains evolve, they tend to be rapidly integrated into multidomain architectures.

**Fig. 4.—:**
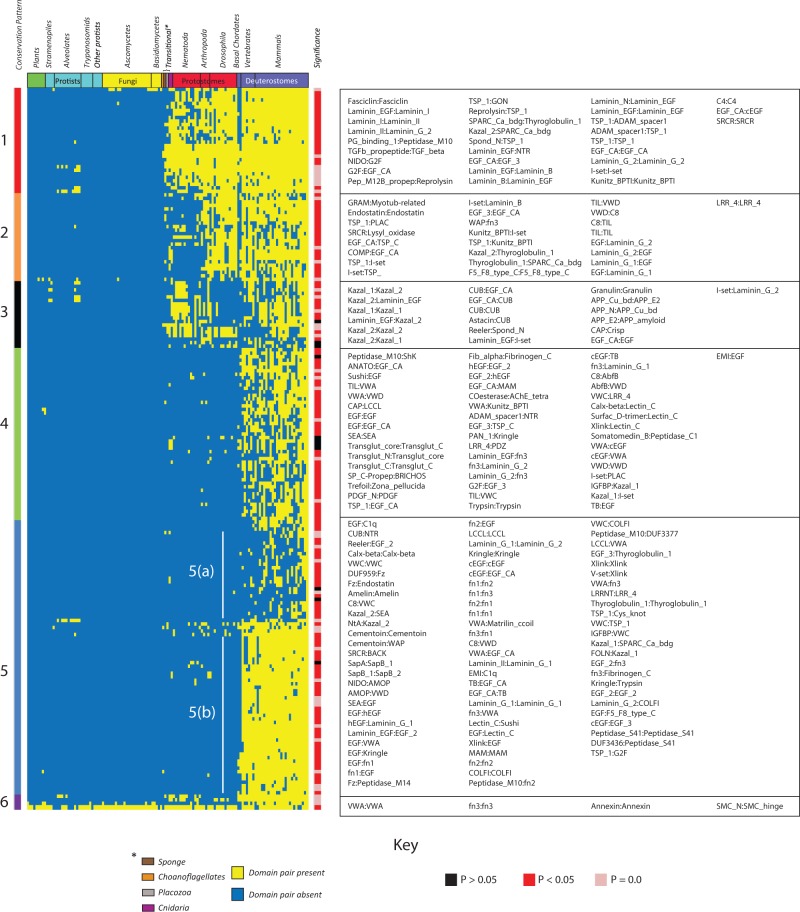
Conservation of ECM domain pairs across 131 species showing the relative abundance of novel, vertebrate-specific domain arrangements. A yellow tile indicates the presence of a specific domain pair whereas blue denotes absence. Species are arranged according to established phylogenetic relationships and domain pairs are hierarchically clustered (city block method, average linkage) according to their conservation pattern. The majority of domain pairs are found significantly more frequently than in a randomized model of domain pair propagation (right hand color track). See also supplementary spreadsheet S18, Supplementary Material online. For domain pairs not conserved in humans see supplementary figure S7 and spreadsheet S21, Supplementary Material online.

To explore this further, we examined the incidence of human ECM domain pairs across 131 eukaryotes (fig. 4 and supplementary spreadsheet S18, Supplementary Material online). Although 28 of 144 domains precede the emergence of metazoans, with a single exception involving SMC3, all domain pairs appear unique to metazoans. SMC3, a component of the cohesin complex, is involved in spindle pole assembly. However, posttranslational addition of chondroitin sulfate gives rise to the secreted proteoglycan bamacan, an abundant basement membrane protein (Shimizu et al. 1998; Ghiselli et al. 1999). More than half of ECM domain pairs found in humans were restricted to vertebrates (127 of 205 [62%], fig. 4) and these included a highly conserved set of domain pairs (Group 5b), together with domain pairs that are less widely conserved (Groups 4 and 5a). The latter implies that in addition to a core-conserved matrix, flexibility in domain pairings may account for lineage-specific innovations.

We next examined the source of domains driving new combinations, categorizing domains as eukaryotic (E), metazoan (M) or vertebrate (V) in origin (fig. 1). In general, the frequency of the observed pair combinations closely matched the expected frequency of a binomial distribution (*P* > 0.05 chi-square goodness of fit test). Of the 90 vertebrate-specific domain pairs 34 (37.8%) involved at least one vertebrate domain of which, only 7 (7.8%) were comprised of two vertebrate domains (V:V). In contrast, 75% (67/90) of vertebrate-specific pairs involve at least one domain of metazoan origin (supplementary fig. S6 and spreadsheets S19–S20, Supplementary Material online). Noteworthy, eukaryotic domain pair combinations (E:E) were significantly enriched (*P* < 0.0005, chi-square goodness of fit test), highlighting the capacity of even ancient (and presumably already well-sampled) domains to contribute to new functional contexts. These findings suggest that emergence of novel ECM domain combinations, which help drive lineage specific innovations, are not dependent on domains of recent origin. Rather, they arise through sampling existing domains irrespective of their age. We next explore whether related domain architectures comprise functionally relevant modules within the ECM network.

### Network Analyses of Domain Adjacency Reveal Domain-Based Functional “Modules” That Display Clade-Specific Rewiring

**Fig. 5.—:**
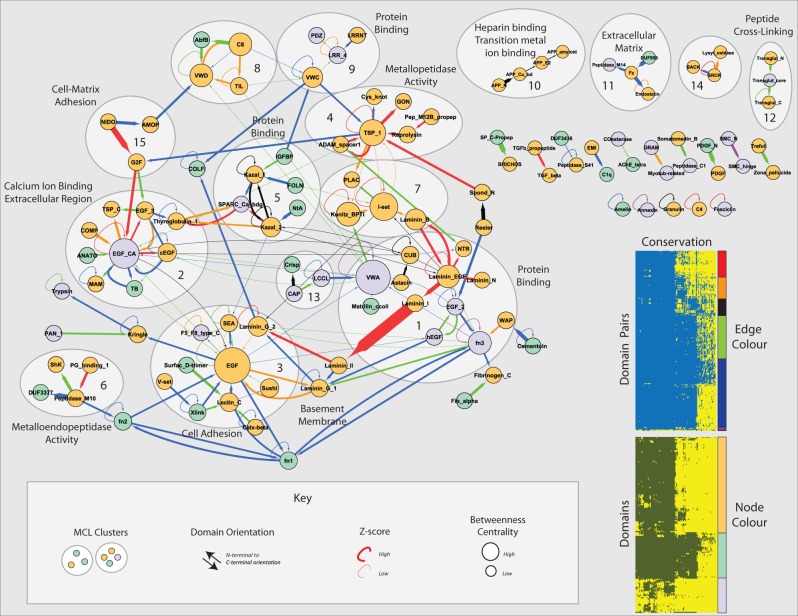
A domain adjacency network revealing substantial extension and rewiring of domain relationships across clades and domain age categories. Directed edges represent adjacency of domains (nodes) in the indicated N-terminal to C-terminal orientation. The statistical significance of each domain pair was generated through comparisons with randomly constructed proteomes (see Materials and Methods) and used to weight each edge by resultant *z*-scores. Note that despite the appearance of thinner edges (due to scaling), the majority of real domain pairs occur significantly more frequently than in randomized simulations. Edges are colored according to domain pair conservation groups defined in figure 4 (upper inset). Node colors correspond to domain age categories as defined in figure 1 (lower inset). MCL clusters representing putative domain modules are numbered and encircled for emphasis. Node size is proportional to betweenness centrality.

To examine functional relationships between domain pairs and how these relationships have changed across different lineages, we constructed a domain adjacency network comprising 117 nodes (domains) connected by 201 edges (fig. 5) representing the domain architectures of all human ECM proteins. Of 205 domain pairs found in humans, 74 appeared to be conserved across metazoans. Over 100 domain pairs are recent additions in vertebrates with a further 25 specific to mammals.

Clustering the network (see Materials and Methods), revealed 15 putative domain modules consisting of three or more domains. Exploiting a previously generated set of PPI-based module annotations (Cromar et al. 2012) together with Gene Ontology mappings, we identified 11 modules statistically enriched for biological process terms (*P* < 0.005—see Materials and Methods, fig. 5). These include: Calcium binding (module 2); cell adhesion (module 3); and metallopeptidase activity (module 4). However, the association of several modules with uninformative terms, such as “protein binding” demonstrates the challenges of this approach. Such limitations are likely due to: 1) incomplete overlap between domain-based and protein-based modules; 2) limited annotation coverage associated with ECM proteins; and 3) biases in functional annotation schemes toward protein-based rather than domain-based annotations (Vogel et al. 2005). Nevertheless, domain modules broadly reflect matrix-associated functional themes.

Within these modules we observe expansion of vertebrate-specific pairs, extending the variety of ECM domain architectures conserved with protostomes. For example, module 3 (cell adhesion) transitioned from a core comprised of EGF and laminin domains to a larger module in which the EGF domain serves as a central hub for a variety of ECM-based architectures. Vertebrate-specific domains such as FN1 and FN2 form further connections (with modules 1, 3, and 6) associated with matrix remodeling and protein binding. Vertebrate-specific domain pairings are also responsible for the emergence of new modules (e.g., modules 9, 11, and 12).

In addition to novel links, the network displays rewiring of domain relationships across evolution. For example, modules 2, 7, 8, 11, and 14 are generally well-conserved with arthropods but many domain relationships are not present in basal metazoans or nematodes. Conversely, several domain pair relationships appear to have been lost in arthropods (e.g., module 13 and between modules 1 and 2) indicating either sequence divergence, loss of function provided by the domain combination or recruitment of additional proteins to replace the function. Apparent losses include a number of domain combinations involving Kazal, Laminin, and CUB domains. Importantly, it is the unique combination of these domains that has been lost rather than the domains themselves. Serine protease inhibitors (Serpins) in which kazal domains are found are involved in protection against autophagy in metazoan digestive systems (Chera et al. 2006). Although homologs have been found in insects they appear to be highly specialized and in some cases structurally diverse (Nirmala et al. 2001; van Hoef et al. 2011).

Among orthologs of human ECM proteins are a large number of poorly conserved, species-specific domain pairs (305/510), suggesting the recruitment or shuffling of domains (supplementary fig. S7 and spreadsheet S21, Supplementary Material online). Further, since a diversity of compatible domain architectures exist for many proteins, individual species represent only a fraction of the possible inventory of domain pairs consistent with a particular function.

Our evolutionary analyses suggest complex patterns of rewiring contributed to clade-specific differences in the usage of otherwise conserved domain pairs. However, conservation is not limited to pairs of domains but may extend to HOODs (e.g., triplets or quadruplets of domains) (Todd et al. 2001; Bashton and Chothia 2002; Kummerfeld and Teichmann 2009). In the next section, we consider HOOD architectures as potential units of selection.

### Patterns of ECM Domain Usage Extend to Conserved Higher-Order Architectures

**Fig. 6.—:**
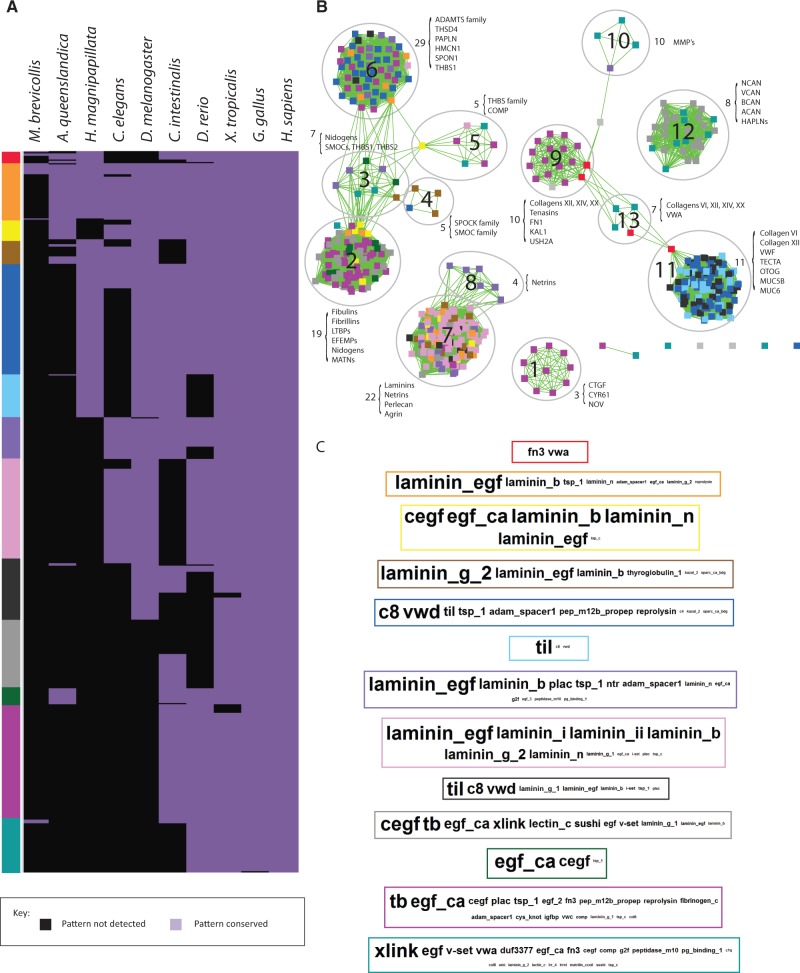
Evidence of accretion and loss of HOODs and their occurrence within multidomain ECM proteins. (*A*) Conservation of HOODs across ten representative species. Domains patterns were hierarchically clustered (Euclidean method, average linkage), and species were arranged according to known phylogenetic relationships. Sequential patterns were defined using the PrefixSpan algorithm and represent combinations of up to four domains occurring in three or more proteins (see Materials and Methods). (*B*) Clusters of related domain patterns. Nodes represent patterns and edges represent shared domains. Node colors relate patterns to their conservation profile (*A*) and to specific domains (*C*) whose relative frequency of occurrence in HOODs within groups is shown as a series of WordClouds (Oesper et al. 2011). (*C*) The relative abundance of domains in specific conservation groups is represented by the font size in each WordCloud. Domain names are ordered by decreasing abundance (i.e., the order is not reflective of specific architectures). WordClouds are surrounded by a colored border linking them to the correspondingly colored conservation group (*A*) which is also consistent with the pattern colors in clusters (*B*). See also supplementary spreadsheets S24–S29, Supplementary Material online.

To identify recurring HOOD architectures within the human ECM we used PrefixSpan (Jian Pei et al. 2001), a sequential pattern mining algorithm, to detect frequent sequential patterns (see Materials and Methods). We define a frequent sequential pattern as an ordered (although potentially discontinuous) set of domains identifiable in at least three proteins. For example, the sequential pattern (A,B,C) can be found in proteins with domain architectures: (A,B,C,D), (X,A,B,C), (Y,A,Y,B,C), (X,Y,A,Z,B,B,C). We identified 588 patterns of which the occurrences of 510 were determined to be statistically significant (*P* < 0.05, Bootstrap resampling; supplementary spreadsheet S22, Supplementary Material online). Among 490 patterns which were most significant (*P* < 0.005), 256 were comprised of four domains while 150 were comprised of three domains (supplementary spreadsheet S23, Supplementary Material online). For each pattern, we examined its conservation across ten representative metazoans (fig. 6*A*) and supplementary spreadsheet S24, Supplementary Material online).

Although there were a number of lineage-specific pattern losses in worm, only a single fly specific loss was observed. The former include patterns involving domains TIL, C8, and VWD, which occur in the human proteins: VWF, TECTA, OTOG and two mucins, MUC5A and MUC6. Consistent with the missing domain patterns, despite worm possessing homologs for at least some of these proteins, the C8 domain appears to have been lost within the nematode lineage (fig. 1). Interestingly, patterns based on these domains are similarly missing in fish though this is likely related to an inability to detect orthologs of VWF, TECTA, OTOG, and MUC6 in this lineage. VWF is an essential clotting factor known to be present in teleosts (Martins et al. 2009) and it is likely that this protein is present but divergent from the human reference sequence. Orthologs of TECTA were detected in other fish lineages, again suggesting difficulty in detecting the *D. rerio* ortholog.

To examine potential overlap in patterns arising from similar domain architectures, we constructed an enrichment map in which nodes represent discrete patterns and links indicate sharing of common domains (fig. 6*B*) and supplementary spreadsheets S25–S29, Supplementary Material online). We identified 13 pattern groups (PGs) whose members share similar domain composition. The relative frequency of specific domains within each PG is shown in figure 6*C*. Rather than PGs being composed of a mosaic of all conservation groups, each PG reflected a limited set of conservation groups. For example, PGs 1, 5, and 12 are composed of patterns associated with vertebrates, whereas patterns in PG 8 are associated with protostomes and deuterostomes. The four largest PGs are associated with a larger range of conservation groups, suggesting expansion of patterns for these groups within distinct lineages. For example, PG 2 includes patterns associated with basal metazoans, together with those that later emerged in the vertebrate lineage, highlighting the ability of apparently fixed domain architectures to acquire new domains that may help drive lineage-specific adaptations.

Mapping HOODs onto the network of domain adjacency revealed a correspondence between HOODs and modules defined on the basis of domain pairs (supplementary fig. S8, Supplementary Material online). This correspondence suggests that HOODs, like their component pairs, cluster around common functional themes, validating the inherent assumptions underlying prior studies of domain pairs. Our study of domain patterns has revealed that HOOD architectures exist and are conserved across multidomain ECM proteins.

## Discussion

The ECM is a defining feature of metazoans consisting of secreted proteins that self-assemble into a complex meshwork of fibers. Connected, they provide essential structure, as well as a platform to organize and translate mechanical and chemical signals into a complex body plan. In a recent survey of 357 genes comprising the core ECM network, approximately two-thirds of the components of the ECM were found to represent recent vertebrate-specific innovations (Cromar et al. 2012). Because domains represent independently folding three-dimensional units of selection, here, we examined the contribution of domain architecture to ECM innovation.

The creation of multidomain proteins has accelerated in the metazoan lineage resulting in a rich diversity of domain architectures (Ekman et al. 2007). Compared with other human proteins, ECM proteins are significantly enriched in multidomain architectures, highlighting the importance of domains in driving the evolution of the ECM. Previous studies of multidomain proteins suggest domain arrangements occur largely through gene fusion, repeat expansion and subsequent domain loss that preferentially occurs at the termini (Bornberg-Bauer and Alba 2013). With the availability of a well-curated data set (Cromar et al. 2012), we examined whether such a model extends to ECM proteins, or if instead other factors have shaped the evolution of the ECM.

Based on their conservation across 131 eukaryotic species, we infer that the emergence of the ECM involved the recruitment of extant domains together with the innovation of new domains and new domain combinations. Subsequent evolution of ECM proteins was driven by domain gain with evidence of rare, clade-specific losses, and rarer domain rearrangements. The recruitment of domains to the ECM appears to have been highly selective, with 109 of 144 being significantly enriched in ECM proteins including 35 exclusively to the ECM. Interestingly, these 109 domains appear to have arisen throughout the evolution of eukaryotes suggesting an ongoing recruitment of domains and associated accumulation of novel functions. The relative rarity of domains of vertebrate origin compared with those of more ancient origin is consistent with previous reports of domain age in which only 12.3% (426 of 3,465 domains) of all human domains were considered to be vertebrate in origin (Toll-Riera and Alba 2013). At the same time, compared with other human proteins, ECM proteins appear to be enriched in vertebrate-specific domains at 24.3% (35 of 144). We therefore conclude that novel domains were important in establishing new ECM functions.

Although we acknowledge that these findings may be impacted by our reliance on domain detection algorithms that may fail to identify divergent members (e.g., not detected in our study were insect serpins, which are known to be highly divergent from their human counterparts) (Nirmala et al. 2001; van Hoef et al. 2011), there is no indication that ECM domains display a wider spectrum of diversity than other domains and hence comparisons between ECM proteins with non-ECM proteins remain. Similarly, domain detection is predicated on the underlying detection of orthologs. Here, we rely on the established Inparanoid pipeline (Berglund et al. 2008), previously shown to outperform other ortholog prediction methods (Chen et al. 2007). Due to the number of proteins involved in initial searches, potentially more sensitive but computationally intense, tree-based approaches were deemed unsuitable for the rapid determination of orthologs across entire proteomes. However, the use of tree-based methods (e.g., Notung [Durand et al. 2006; Stolzer et al. 2012]) would be recommended for the resolution of more complex gene families in future studies. The appearance of PDZ within our ECM domain data set was unexpected and due to the inclusion of ERBB2IP, a protein annotated in the Gene Ontology (Ashburner et al. 2000) as a component of basement membrane. However, although likely to be functionally related, a review of the cited reference (Borg et al. 2000) suggests the annotation should be amended to “basolateral membrane.” Given the necessarily iterative nature of curation efforts, this example highlights an important role of systematic studies in helping to validate functional assignments in large, publicly available data sets.

Recent studies probing the mechanisms of de novo domain creation and recombination suggest that the majority of vertebrate-specific (i.e., new) domains first emerged as single-domain proteins (Toll-Riera and Alba 2013). However, we found that most de novo domain gains in ECM proteins take place in the context of existing proteins or as fusions rather than as singleton domains in new genes. Novel, tetrapod-specific domains, for example, were found either exclusively in singleton or exclusively in multidomain proteins implying rapid recruitment of new domains into multidomain architectures. We therefore suggest that given the highly interconnected nature of the ECM, in which many proteins physically interact, the emergence and subsequent recruitment of new domains occurs under unique selective pressures that drive their integration into existing multidomain architectures.

Proteins involved in extracellular structures were previously found to be enriched in promiscuous domains (Basu et al. 2008). However, given that ECM proteins are mainly composed of domains either enriched for or specific to ECM proteins, it is unlikely that they are highly promiscuous. Indeed, we found the majority of ECM-associated domains were characterized by low frequency and promiscuity, with only a small fraction of ECM domains displaying high promiscuity. Existing domains appear to be continually recruited during evolution of the ECM; a process favoring domains with the highest frequency (i.e., older domains) (Apic et al. 2003; Basu et al. 2008). It is reasonable to hypothesize, therefore, that more ancient domains are likely to play a dominant role in ECM organization. Consistent with this, we found ECM hubs to be significantly enriched in highly promiscuous domains.

Although it is likely that the emergence of novel domains played an essential role in the establishment of the matrix, we found that within the 144 ECM-associated domains, only about a quarter were of vertebrate origin. This suggested that for the two-third of human ECM proteins lacking detectable homologs outside vertebrates, subsequent evolution of the ECM was likely driven through mechanisms other than domain innovation. Focusing on domain cooccurrence, we found that most (67/90) vertebrate-specific domain pairs involved at least one domain of more ancient origin, suggesting that ECM innovation in vertebrates was largely driven through the generation of novel domain combinations. Furthermore, while the ECM is enriched in vertebrate-specific domains, pairwise patterns of domain age were consistent with a random model of domain propagation: New domains participate in a continuous process of random domain assortment and new functions arise as much from novel domain combinations as from new domains themselves (Vogel et al. 2005). As an aside, we did note that older domain combinations composed of two domains of premetazoan origin were the only pairs statistically overrepresented in ECM proteins (*P* < 0.0003). Such preferential recruitment might be explained through the potential impact of novel and consequently disordered domains disrupting existing biological functions; established domains, by their very nature, being less likely to cause such disruptions (Moore and Bornberg-Bauer 2012).

The dependency on new domains to provide novel innovations in vertebrates is further minimized through the reuse of domains either through tandem duplications or reordering resulting in new functions (Bashton and Chothia 2002). Domain repeats are often expanded through duplications of several domains at a time, facilitated in ECM by domains being encoded as single exons (Bjorklund et al. 2006; Hynes 2012). Accordingly, tandem repeats accounted for the most frequent ECM domain pairs. The preferential enrichment of these repeats within structural proteins supports previous suggestions that domain repeats are driven by large structural complexes (Apic et al. 2001). Beyond tandem duplications, ECM domain pairs appear bidirectionally (in both a forward (A-B) and reverse (B-A) orientation) more frequently than expected. Excluding 41 identical pairs, 12.8% (21 of 164) human ECM domain pairs were bidirectional compared with previous estimates of 3–6% for all proteins (Kummerfeld and Teichmann 2009). Interestingly, previous studies (Todd et al. 2001; Bashton and Chothia 2002) suggest forward and reverse domain arrangements result in different functions, supporting the notion that such arrangements reduce the reliance on generating novel domains to drive innovation.

To further elucidate origins of domain-pairs, we applied a network-based approach to identify domains of eukaryotic and metazoan origin recruited into vertebrate-specific combinations. This network revealed a surge of vertebrate innovation, driven by the acquisition of novel domain arrangements. Although many aspects of vertebrate skeletal evolution remain unclear (Donoghue and Sansom 2002; Donoghue et al. 2006), the identification of several domain combinations appearing after the split between teleosts and tetrapods suggest a potential role in the evolution of skeletal tissues during the transition to life on land. We also identified an additional 305 ECM domain pairs, absent from humans and poorly conserved elsewhere, indicating that other metazoan lineages have acquired their own complements of novel domain combinations (supplementary fig. S7 and spreadsheet S21, Supplementary Material online).

An important contribution of this study is the application of sequential pattern mining as a method to investigate HOOD architectures and their conservation. Vogel et al. previously suggested that contiguous two and three domain combinations can result in evolutionary conserved three-dimensional structures, termed “supra-domains” (Vogel et al. 2004). Subsequent studies further showed that domains do not necessarily need to be contiguous in order to contribute to a conserved three-dimensional fold (Uliel et al. 2001; Fliess et al. 2002; Bornberg-Bauer and Alba 2013). Our analyses revealed that HOODs accumulate across orthologs, gradually increasing the complexity of domain architectures; a general process some have termed “accretion” (Koonin 2000). These are accompanied by clade-specific losses suggesting that, as for domains and domain pairs, HOODs represent units driving evolutionary change. For example, loss of patterns involving TIL, C8, and VWD domains in nematodes correlate with fewer paralogs of VWF, MUC5A, and TECTA proteins, together with the absence or divergence of OTOG and MUC6 proteins in this lineage.

The emergence of metazoan life involved the innovation of a large number of novel ECM domains. Vertebrates subsequently exploited these domains through the generation of novel domain combinations to yield systems such as a biomineralized skeleton, a network of elastic fibers and a variety of organ systems supported by an array of specialized matrices.

## Conclusions

Consistent with the current consensus model of domain evolution in which the accretion of domains and domain combinations and selective losses lead to increasingly complex, multidomain architectures, our study has shown that the major driving force for human ECM evolution has been the innovation of novel domain combinations, rather than novel domains. These domain combinations, which we have extended to include HOODs, have evolved to support the unique, dual roles of the ECM in structure and signaling. We identified specific domains of eukaryotic, metazoan, and vertebrate origin which, independent of their age, gave rise to clade-specific domain combinations. However, the prevalence of older domain pairs among the large number of vertebrate-specific pairs suggests the mechanism of novel domain acquisition by ECM proteins may be different than other proteins; dominated by domain fusion and the recruitment of additional domains to existing architectures rather than the de novo creation of independent domains. This study reveals that the organization of the matrix is mediated by a relatively small number of highly promiscuous domains which are enriched in structural proteins that have emerged as the basis for network hubs. Together these results emphasize the importance of validating models derived from global domain analyses, through focusing on specific biological processes and/or specific classes of proteins.

## Supplementary Material

Supplementary figures S1–S8 and excel spreadsheets S1–S29 are available at *Genome Biology and Evolution* online (http://www.gbe.oxfordjournals.org/).

Supplementary Data
