# Supplementary material for: New Tricks for “Old” Domains: How Novel Architectures and Promiscuous Hubs Contributed to the Organization and Evolution of the ECM
Source: Genome Biol Evol. 2014 Oct 15;6(10):2897–917. doi: 10.1093/gbe/evu228 (PMC4224354; doi:10.1093/gbe/evu228)
Supplement: Supplementary Data [file supp_6_10_2897__index.html]

New tricks for ‘old’ domains: How novel architectures and promiscuous hubs contributed to the organization and evolution of the ECM. — New Tricks for “Old” Domains: How Novel Architectures and Promiscuous Hubs Contributed to the Organization and Evolution of the ECM — Supplementary Data 

# New Tricks for “Old” Domains: How Novel Architectures and Promiscuous Hubs Contributed to the Organization and Evolution of the ECM

## Supplementary Data

files

**Files in this Data Supplement:**

- Supplementary Data - pdf file
- Supplementary Data - xlsx file
