# Supplementary material for: New Tricks for “Old” Domains: How Novel Architectures and Promiscuous Hubs Contributed to the Organization and Evolution of the ECM
Source: Genome Biol Evol. 2014 Oct 15;6(10):2897–917. doi: 10.1093/gbe/evu228 (PMC4224354; doi:10.1093/gbe/evu228)
Supplement: Supplementary Data [file supp_evu228_Supplementary_Figures_S1-S8.pdf]

Supplementary Figure

- eukaryotes (131 nodes)
  - animals (67 nodes)
    - chordates (33 nodes)
      - vertebrates (31 nodes)
        - mammals (24 nodes)
          - placentals (22 nodes)
            - primates (5 nodes)
            - rodents (4 nodes)
            - carnivores (2 nodes)
            - insectivores (2 nodes)
            - rabbits & hares (2 nodes)
            - bats (1 node)
            - even-toed ungulates (1 node)
            - odd-toed ungulates (1 node)
            - other placentals (4 nodes)
          - monotremes (1 node)
          - marsupials (1 node)
        - bony fishes (5 nodes)
        - frogs & toads (1 node)
        - birds (1 node)
      - tunicates (2 nodes)
    - insects (17 nodes)
      - flies (15 nodes)
        - mosquitos (3 nodes)
        - other flies (12 nodes)
      - bees (1 node)
      - moths (1 node)
    - nematodes (13 nodes)
    - cnidarians (2 nodes)
      - sea anemones (1 node)
      - hydrozoans (1 node)
    - sponges (1 node)
    - placozoans (1 node)
  - fungi (29 nodes)
    - ascomycetes (22 nodes)
    - basidiomycetes (6 nodes)
    - microsporidians (1 node)
  - apicomplexans (11 nodes)
  - green plants (8 nodes)
    - flowering plants (5 nodes)
      - eudicots (3 nodes)
      - monocots (2 nodes)
    - green algae (3 nodes)
  - kinetoplastids (4 nodes)
  - oomycetes (3 nodes)
  - diatoms (1 node)
  - choanoflagellates (1 node)
  - trichomonads (1 node)
  - diplomonads (1 node)
  - ciliates (1 node)
  - cellular slime molds (1 node)
  - cryptomonads (1 node)
  - red algae (1 node)
  - other eukaryotes (1 node)

Supplementary Fig. S1: A screen capture from the NCBI Taxonomy Browser illustrating the breadth of the phylogeny used in this study. Each 'node' represents one of the 131 fully-sequenced eukaryotes used for conservation analyses.

Supplementary Figure

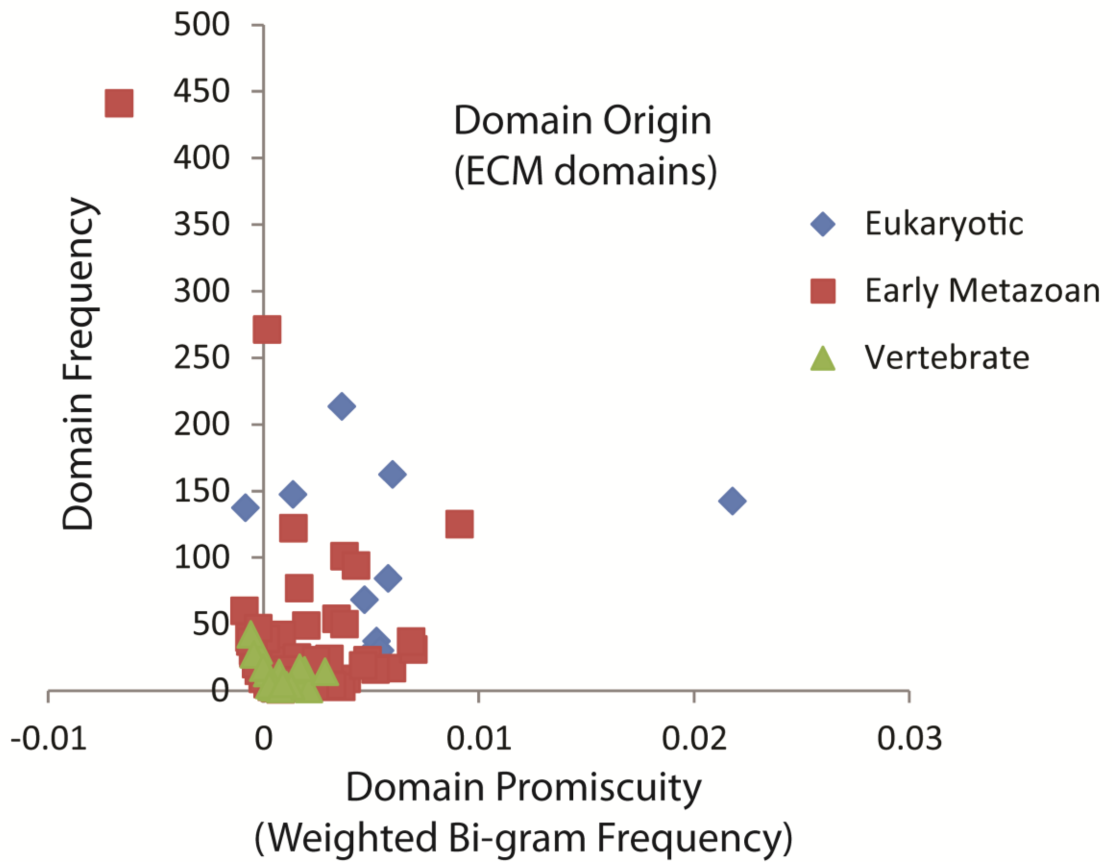

Supplementary Fig. S2: Comparison of domain frequency vs. domain promiscuity (weighted bigram frequency) for human ECM domains of Eukaryotic (blue), Early Metazoan (red) and Vertebrate origin (green).

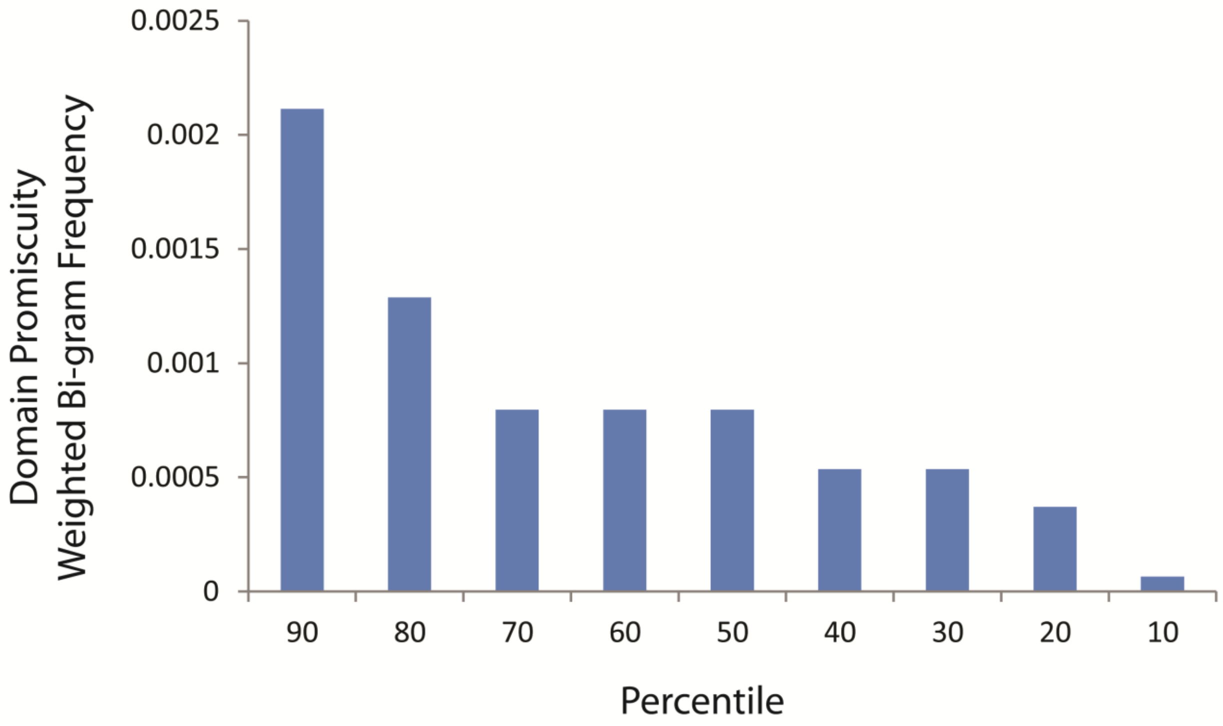

Supplementary Fig. S3: Domain promiscuity score cutoffs for all human Pfam A domains at each percentile. The cutoff score corresponding to the 90th percentile (top 10% of domains ranked by promiscuity scores) corresponding to a weighted bigram frequency > 0.002 was used to classify the threshold for 'high promiscuity' ECM domains (38 of the 124 ECM domains found in multi-domain architectures).

Supplementary Figure

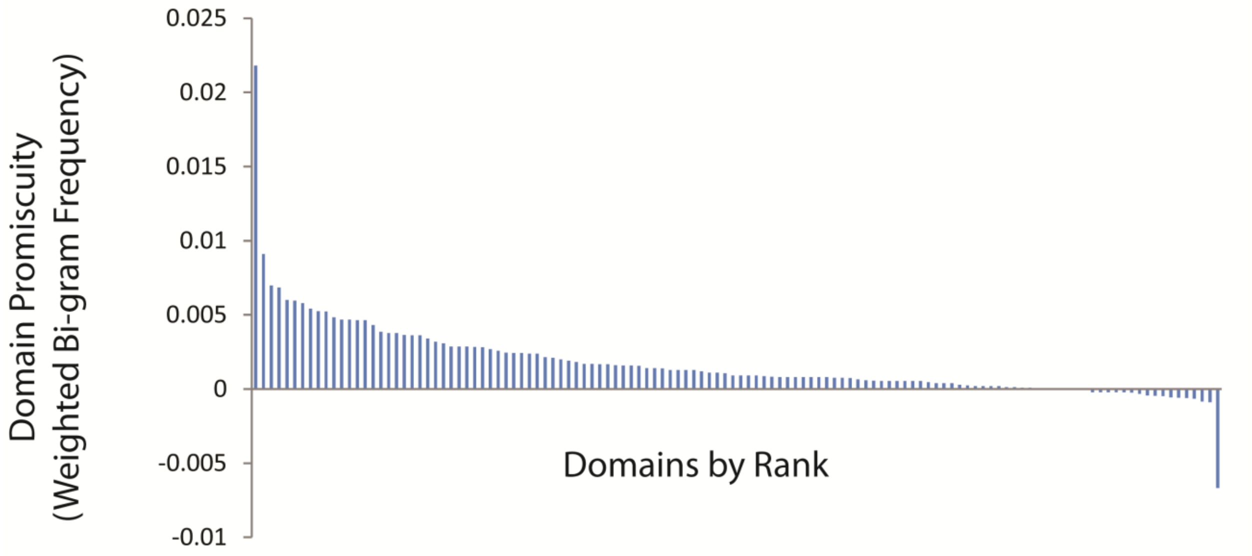

Supplementary Fig. S4: Distribution of promiscuity (weighted bi-gram frequency) for 124 ECM domains appearing in multi-domain architectures.

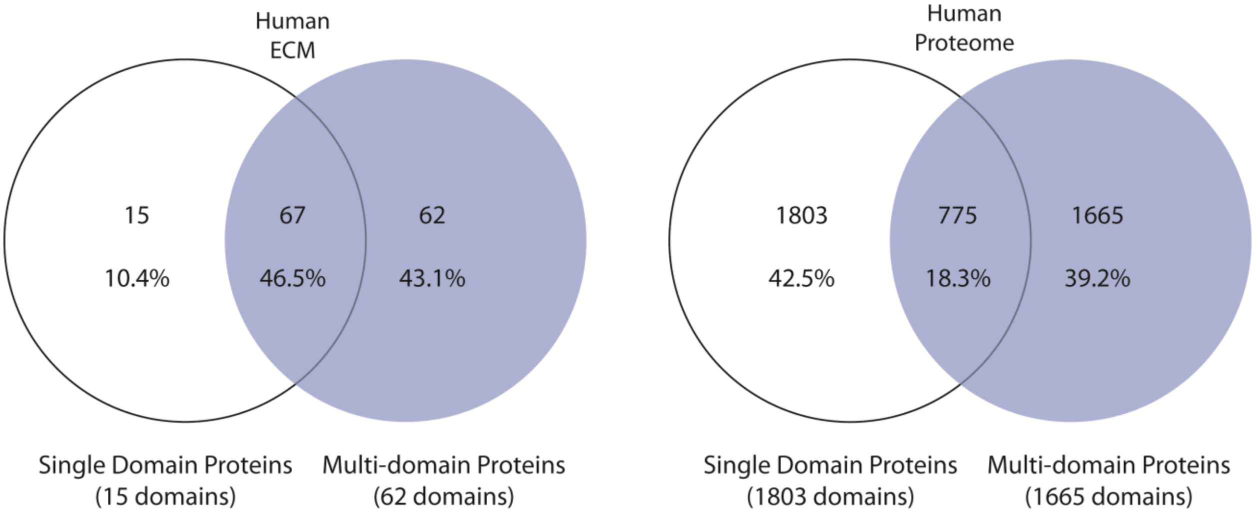

Supplementary Fig. S5: Proportion of Pfam A domains found in single and multi-domain contexts for human ECM proteins vs. all human proteins.

Supplementary Figure

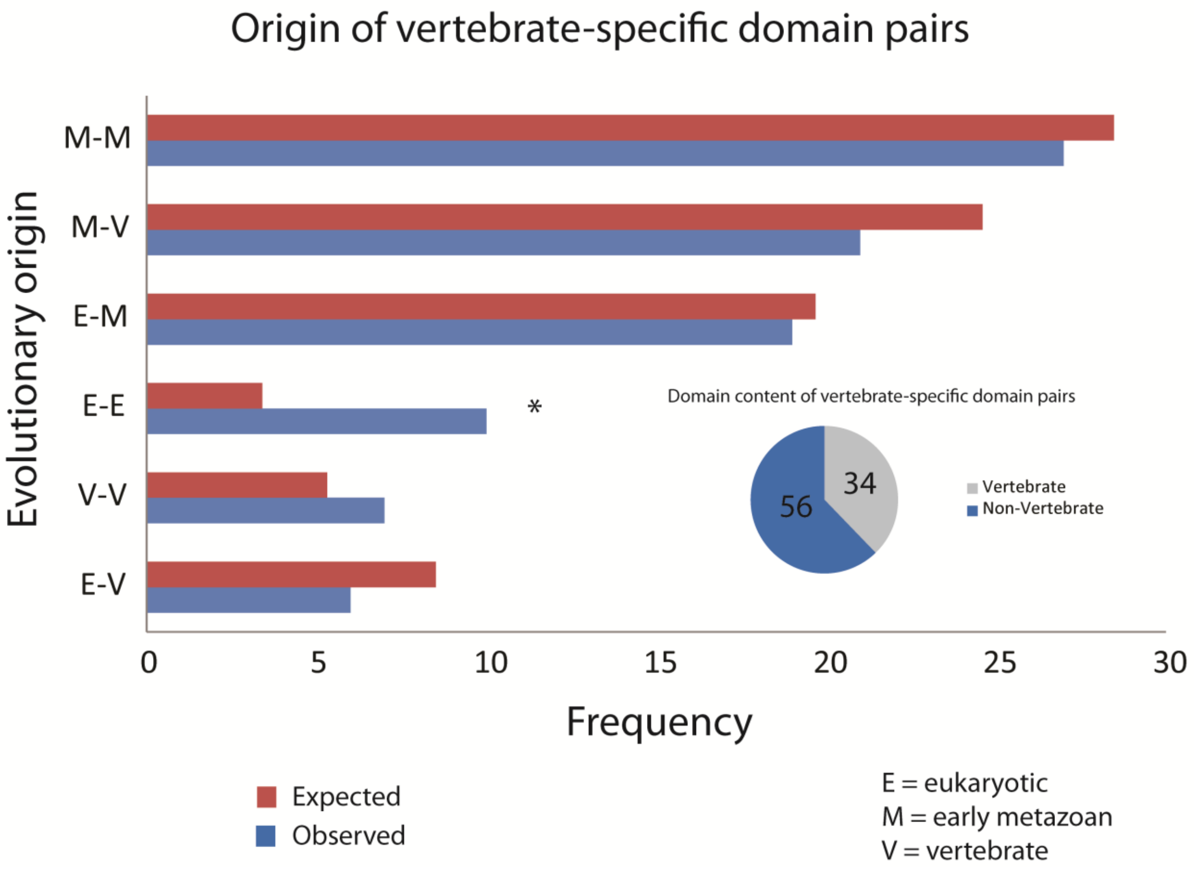

Supplementary Fig. S6: Origin of Vertebrate specific domain pairs. Relative frequency of domain pairs in humans comprised of domains with various combinations of domain origin (blue) versus the expected frequency based on the binomial distribution (red) given the frequency of individual domains in each age category. The asterisk indicates that domain pairs consisting of two eukaryotic domains were observed more frequently than expected and this difference was statistically significant. Vertebrate specific domain pairs consisted of more non-vertebrate than vertebrate domains (inset).

Supplementary Figure

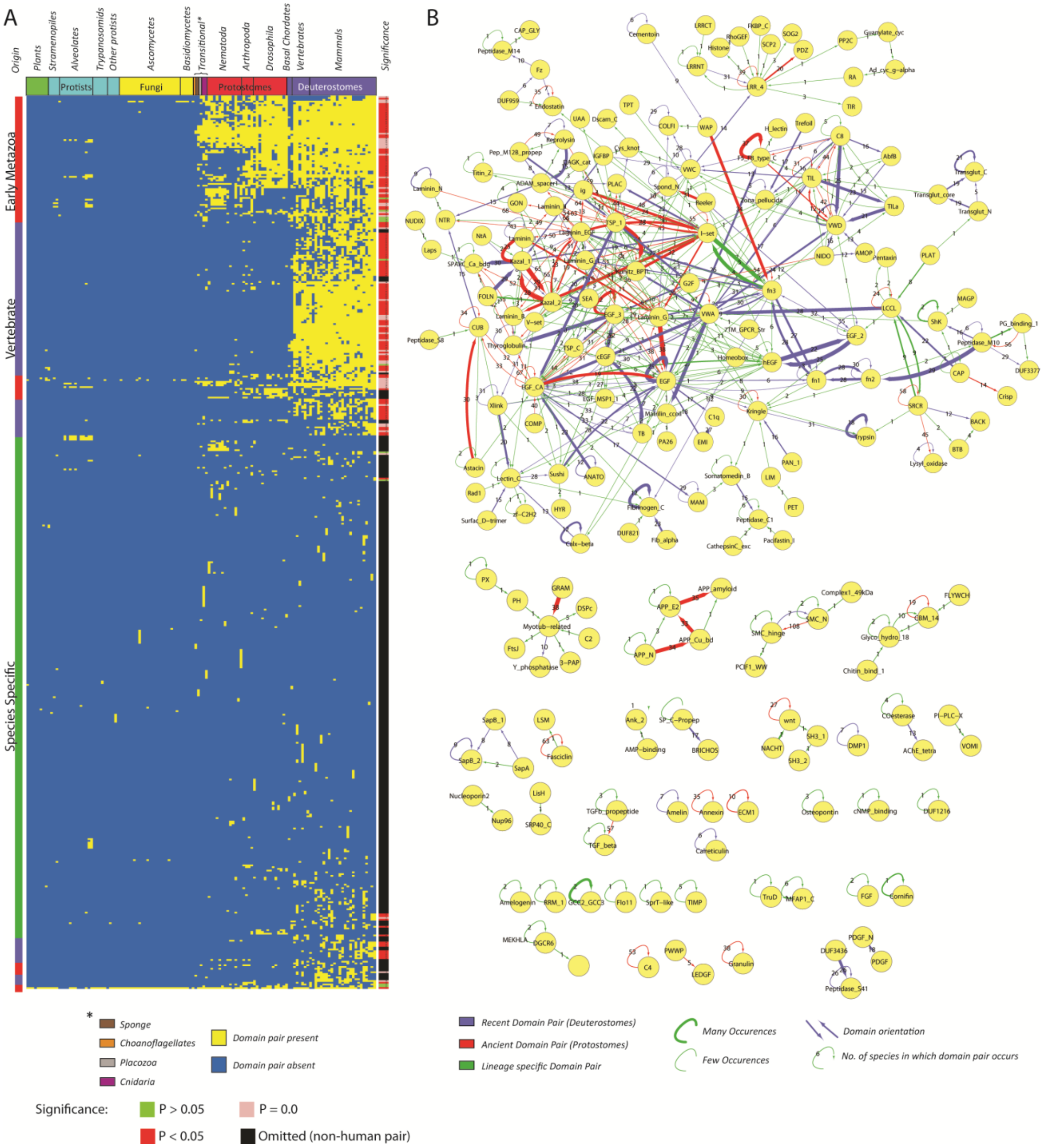

Supplementary Fig. S7: Conservation of ECM domain pairs (all species). A – Domain pairs were broadly categorized as conserved in vertebrates (purple), early metazoa (red) or lineage specific (green). Note that only domain pairs found in humans were included in the statistical simulation to determine the significance of domain pairs. B – Directed network of domain pairs with edge thickness indicating total frequency of domain pairs (all proteins and species) and numbered edges indicating the number of species in which the domain pair occurs. Edges are coloured according to the conservation groups defined in part A.

Supplementary Figure

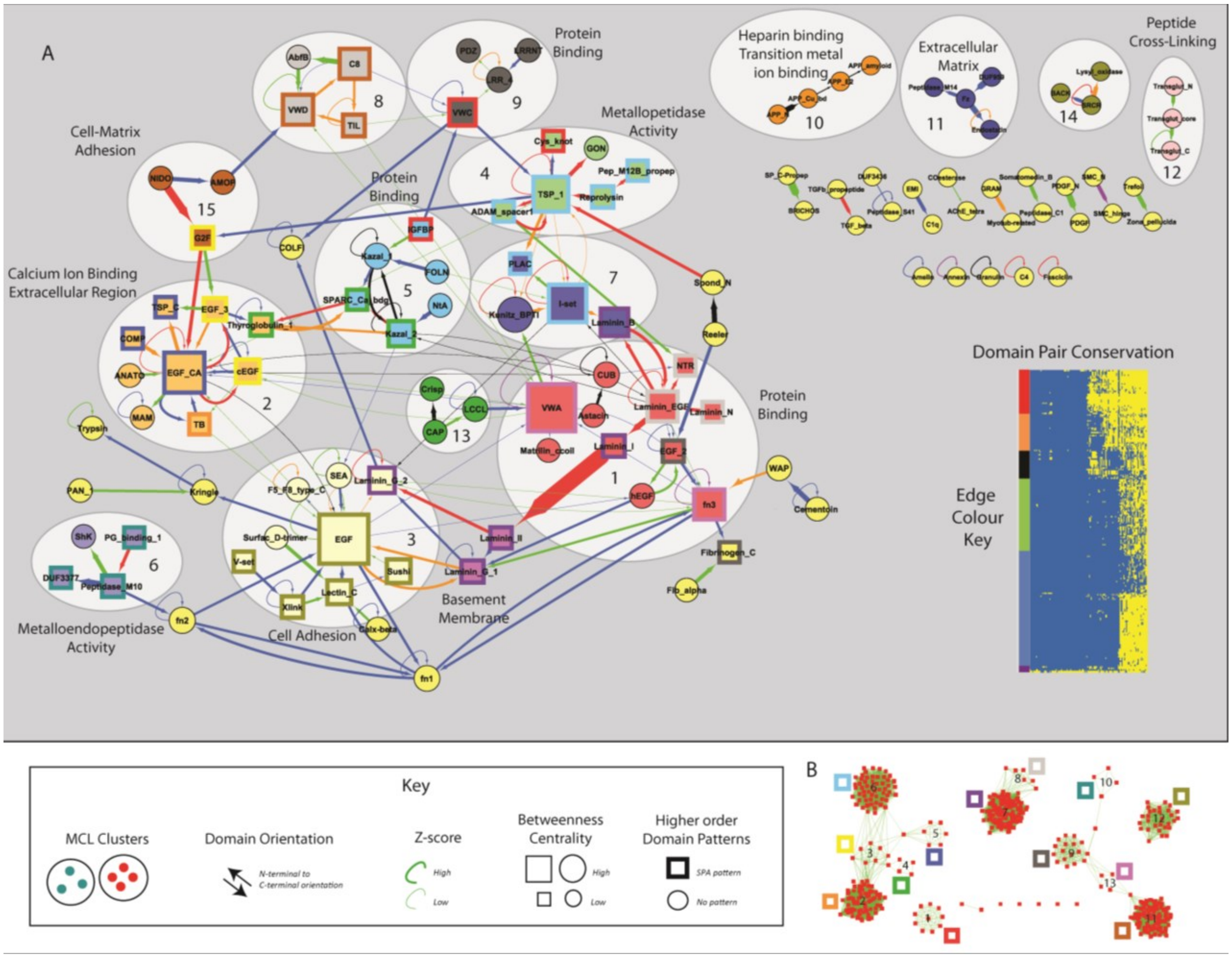

Supplementary Fig. S8: Domain adjacency. A – Directed network of domain pairs with arrows indicating the N to C-terminal arrangement of domains. Edge thickness is proportional to the z-score representing the frequency of each real domain pair relative to its frequency in 10,000 simulated human proteomes (see methods). Edges are coloured according to domain pair conservation groups defined in fig. 4 (also inset). Node colors provide a visual representation of MCL determined clusters representing putative domain modules (numbered and encircled for emphasis). Node size is proportional to betweenness centrality. Square nodes represent domains that are part of higher order domain patterns further defined in fig. 6 with borders color-coded as defined in inset B for quick reference. Note the general agreement between domain-pair based modules and the clustering of higher order domain patterns.

Supplementary Figure
